# A systematic review and meta-analysis of the epidemiological characteristics of COVID-19 in children

**DOI:** 10.1186/s12887-022-03624-4

**Published:** 2022-10-22

**Authors:** Fardis Vosoughi, Rangarirai Makuku, Marcarious M. Tantuoyir, Farbod Yousefi, Parnian Shobeiri, Amirali Karimi, Sanam Alilou, Ronald LaPorte, Curtis Tilves, Mohammad Hossein Nabian, Mir Saeed Yekaninejad

**Affiliations:** 1 Department of Orthopedics and Trauma Surgery, Shariati Hospital, and, School of Medicine, Tehran University of Medical Sciences, Tehran, Iran; 2 Center for Orthopedic Trans-Disciplinary Applied Research (COTAR), Tehran University of Medical Sciences, Tehran, Iran; 3 School of Medicine, Tehran University of Medical Sciences, Tehran, Iran; 4 Department of Epidemiology and Biostatistics, School of Public Health, Tehran University of Medical Sciences, Poursina Avenue, Tehran, Iran; 5 Biomedical Engineering Unit, University of Ghana Medical Center (UGMC), Accra, Ghana; 6 Department of Orthopedic Surgery, Mayo Clinic, Rochester, MN USA; 7 Department of Epidemiology, Graduate School of Public Health, University of Pittsburgh, Pittsburgh, PA USA

**Keywords:** Pediatrics, Epidemiology, COVID-19, SARS-COV-2, Children, Meta-Analysis, Systematic review

## Abstract

**Background:**

Several individual studies from specific countries have reported rising numbers of pediatric COVID-19 cases with inconsistent reports on the clinical symptoms including respiratory and gastrointestinal symptoms as well as diverse reports on the mean age and household exposure in children. The epidemiological characteristics of COVID-19 in children are not fully understood, hence, comprehensive meta-analyses are needed to provide a better understanding of these characteristics.

**Methods:**

This review was conducted in Medline, Scopus, Cochrane library, Embase, Web of Science, and published reports on COVID-19 in children. Data were extracted by two independent researchers and a third researcher resolved disputes. STATA software and the random-effect model were used in the synthesis of our data. For each model, the heterogeneity between studies was estimated using the Q Cochrane test. Heterogeneity and publication bias were calculated using the I^2^ statistic and Egger’s/Begg’s tests.

**Results:**

The qualitative systematic review was performed on 32 articles. Furthermore, the meta-analysis estimated an overall rate of involvement at 12% (95% CI: 9–15%) among children, with an I^2^ of 98.36%. The proportion of household exposure was calculated to be 50.99% (95% CI: 20.80%–80.80%) and the proportion of admitted cases was calculated to be 45% (95% CI: 24%–67%). Additionally, the prevalence of cough, fatigue, fever and dyspnea was calculated to be 25% (95% CI: 0.16–0.36), 9% (95% CI: 0.03–0.18), 33% (95% CI: 0.21–0.47) and 9% (95% CI: 0.04–0.15), respectively. It is estimated that 4% (95% CI: 1–8%) of cases required intensive care unit admission.

**Conclusions:**

The pediatric clinical picture of COVID-19 is not simply a classic respiratory infection, but unusual presentations have been reported. Given the high incidence of household transmission and atypical clinical presentation in children, we strongly recommend their inclusion in research and population-based preventive measures like vaccination as well as clinical trials to ensure efficacy, safety, and tolerability in this age group.

**Supplementary Information:**

The online version contains supplementary material available at 10.1186/s12887-022-03624-4.

## Background

COVID-19 is a highly contagious viral illness which has birthed several mutations thereby making it difficult to bring an end to the current pandemic. According to research, SARS-CoV-2 requires the ACE2 receptor to enter human cells, and ACE2 is found in a variety of organs across the body [1, 2]. An increasing body of research implies that asymptomatic and pre-symptomatic cases of severe acute respiratory syndrome coronavirus 2 (SARS-CoV-2) may significantly contribute to its spread (COVID-19) [3]. SARS-CoV-2 can infect people of all ages and could have long-term consequences for human health. It has a long incubation time, high infectivity, atypical clinical signs, and a significant fatality rate in elderly adults [4, 5]. Although the epidemiology of COVID-19 in adults has been extensively studied, our understanding of the prevalence and consequences of COVID19 in children remains limited. The majority of children infected with SARS-CoV-2 show minimal infection according to an extensive analysis of pediatric cases [6, 7]. Pediatric patients have been suggested as asymptomatic reservoirs of COVID-19 with their burden barely understood due to the mild and asymptomatic nature of the disease [2, 3, 8]. Despite these, few investigations have concentrated on the epidemiological and clinical aspects of pediatric COVID-19 [5].

With current immunization methods focusing mainly on adults, the pediatric population remains vulnerable to COVID-19 infection [9]. Though the unrelenting efforts of scientists have provided the first oral antiviral (Molnupiravir), which has been approved and shows a significant reduction in hospitalization or mortality in mild COVID-19 cases with good safety and tolerability profile in adults, there is little to insufficient data on pediatric cases [10, 11]. Hence, it is critical to be prepared for new waves in terms of pediatric protection and infection control. This could be enhanced by a comprehensive examination of existing pieces of evidence showing illness transmission trends, as well as distinctive and consistent epidemiological aspects of the infection [4, 9, 12].

Previous studies have reported some unique characteristics in pediatric COVID-19 cases such as lower mortality rate, longer incubation period, and longer respiratory and fecal shedding with high RNA load (83%) [13]. Furthermore, potential comorbidities and coinfections particularly in children, have a critical presentation, with infants younger than 6 months having a substantially higher risk of critical illness [2, 14]. Numerous published articles describe children being infected as part of family clusters [13, 15–21], but limited data exists to appraise the cumulative percentage of household exposures before the diagnosis of COVID-19 in children [4]. Exploring these records in any meta-analysis on COVID-19 is of paramount importance.

Hence, this study will attempt to delineate the rate of pediatric involvement in COVID-19, demographic features of the affected children, the proportion of severe cases, and the rate of household exposure as well as future recommendations on pediatric COVID-19 cases based on current epidemiological evidence. It will further address the aspect of clinical presentation, whether affected children present with symptoms of classic viral pneumonia or rather present with gastrointestinal (GI) manifestations.

## Methods

### Overview

This systematic review and meta-analysis were performed per the Preferred Reporting Items for Systematic Reviews and Meta-Analysis (PRISMA) checklist [22]. The completed PRISMA checklist for the current review is available in the supplementary file.

### Search strategy and selection process

**Fig. 1:**
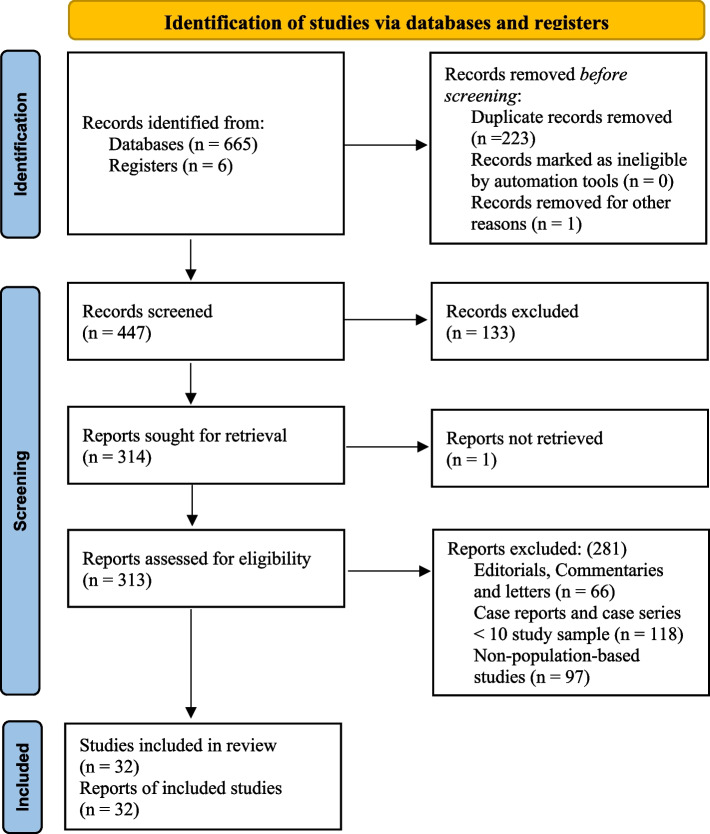
PRISMA flowchart for search strategy

We conducted an extensive search in Medline (indexed by PubMed), Embase, Scopus, Cochrane library, and Web of Science databases on published studies from January 20^th^, 2020 until April 30^th^, 2021. Two independent researchers (F.V. and P.S.) performed a primary search simultaneously, and in cases of difference in opinion, they referred back to the selection protocol. If the dispute remained, a third individual (MSY) made the final decision. Keywords included (Pediatric OR children OR child) AND (COVID-19 OR SARS-COV-2). A revisit to the database was conducted by the first author at 9 AM on May 10^th^, 2021. Furthermore, our data were scrutinized by checking the Worldometer reports [23] and their sources. This investigation included only articles written in English. The process of study selection is fully reported with a PRISMA flow diagram in Fig. 1 of the final report.

### Eligibility criteria

This study includes peer-reviewed published studies from January 20^th^, 2020 until April 30^th^, 2021, including registries [15–17, 24–28], cross-sectional observational studies and case series [13, 18, 29–34], and case reports [19–21, 35–43], which reported children (0–19 years old) affected by SARS-COV-2. Specifically, we included the original and peer-reviewed English papers fulfilling the eligibility criteria in the final report. The following records were excluded in the present study;

• Review articles, editorials, commentaries, opinions, or any studies with no original data.
• Ongoing projects (e.g., articles discussing the protocol of a future study, papers in preprint servers, etc.)
• Non-human (e.g., animal and laboratory) studies.
• Duplicated results in databases.

Furthermore, relevant references to the included studies were annexed. For countries with no relevant published studies, available national registry data were used.

### Critical appraisal

Cross-sectional observational studies that met the aforementioned criteria underwent bias assessment using the Newcastle–Ottawa Quality Assessment Scale (NOS) (Supplementary Table 1 and Fig. 1) [44]. The highest score (which is 16) shows the highest quality of a study considering clear goals, representativeness of the sample, sufficient and justified sample size, having defined the status of the non-respondents, ascertainment of the exposure, high comparability, and independent assessment of outcome with the use of appropriate statistical tests. Studies with a NOS score of 5 or less were excluded from the meta-analysis.

### Outcomes

The main outcomes of interest included a proportion of children among the total infected population, mean age and sex ratio among infected children, and percentages of different clinical symptoms. In addition, the proportion of children with household exposure before diagnosis and the proportion of admitted/severe cases were extracted. Severe cases were defined as those who were admitted into the hospital/intensive care unit with at least SpO2 < 94%, those who had multiple organ failure, septic shock, and respiratory failure.

### Statistical analysis

Meta-analysis was conducted utilizing metaprop and metan commands in STATA software version 15.0 StataCorp, College Station, TX. Forest plots were used to show the point and interval estimation of studies and the overall estimation of all studies. For numeric variables, the mean and standard error were used for the meta-analysis. Median or Mid-range was used instead of mean if it was not available. In most of the studies, standard deviation (SD) was reported and standard error was estimated by ${}^{\mathrm{SD}}\left/{}_{\sqrt{\mathrm{n}}}\right.$ formula. For those studies that reported range or interquartile range, SD was estimated by dividing the range by 2 and the interquartile range by 1.35, and thereafter, the standard error was estimated from SD.

**Fig. 2:**
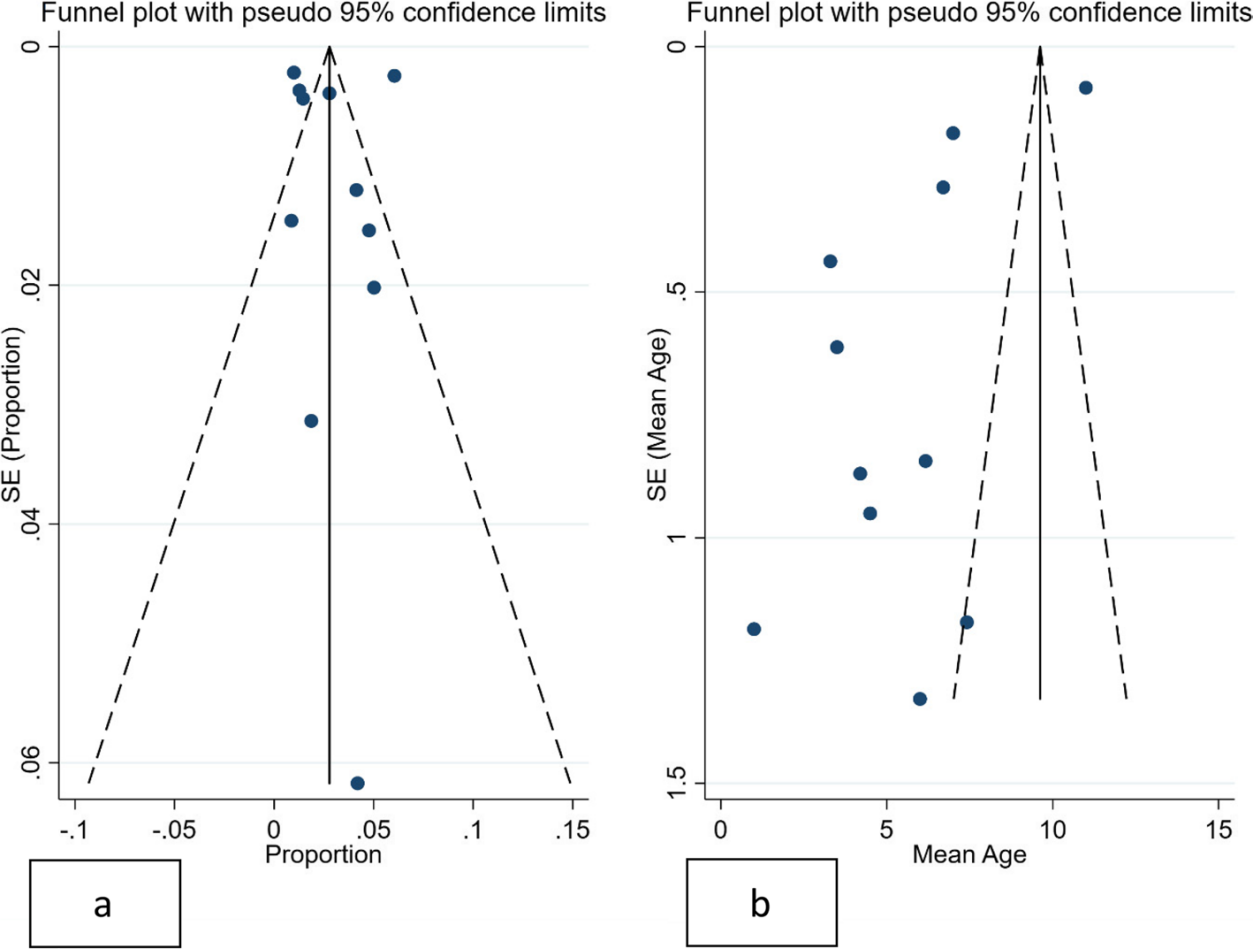
Combined Funnel Plots for demographic features of pediatric COVID-19

Publication bias was evaluated for meta-analyses on 10 papers or more, by using Egger’s, Begg’s tests. *P-values of 0.05* or less were considered significant. Furthermore, publication bias funnel plots for analysis of the proportion of pediatric cases and mean age were drawn (Fig. 2).

Heterogeneity was assessed statistically using the standard Chi-square test. The random-effect model was used and for each model, the heterogeneity between studies was estimated using the Q Cochrane test. Also, the intensity of heterogeneity was calculated using the I^2^ statistic.

## Results

### Study selection

A total of 671 studies were identified after the primary search, of which 224 were removed due to duplication and other reasons. Titles and abstracts of 447 papers were then checked for their eligibility for this study and 134 articles were excluded (Non-English Text (1), Review articles about the infection (62), Erratum: (2), Irrelevant to our study (68), and no full text (1)). Full texts of 308 published articles and 5 available national registry data on pediatric COVID-19 were studied, which resulted in the exclusion of 281 more articles (Fig. 1). The qualitative systematic review was performed on 32 articles. We did not import low-quality articles, fortunately, we did not have any. Analytically, 27 articles were of good quality 79.4% and the other 7 articles were Fair 20.6%.

### Study characteristics

**Fig. 3:**
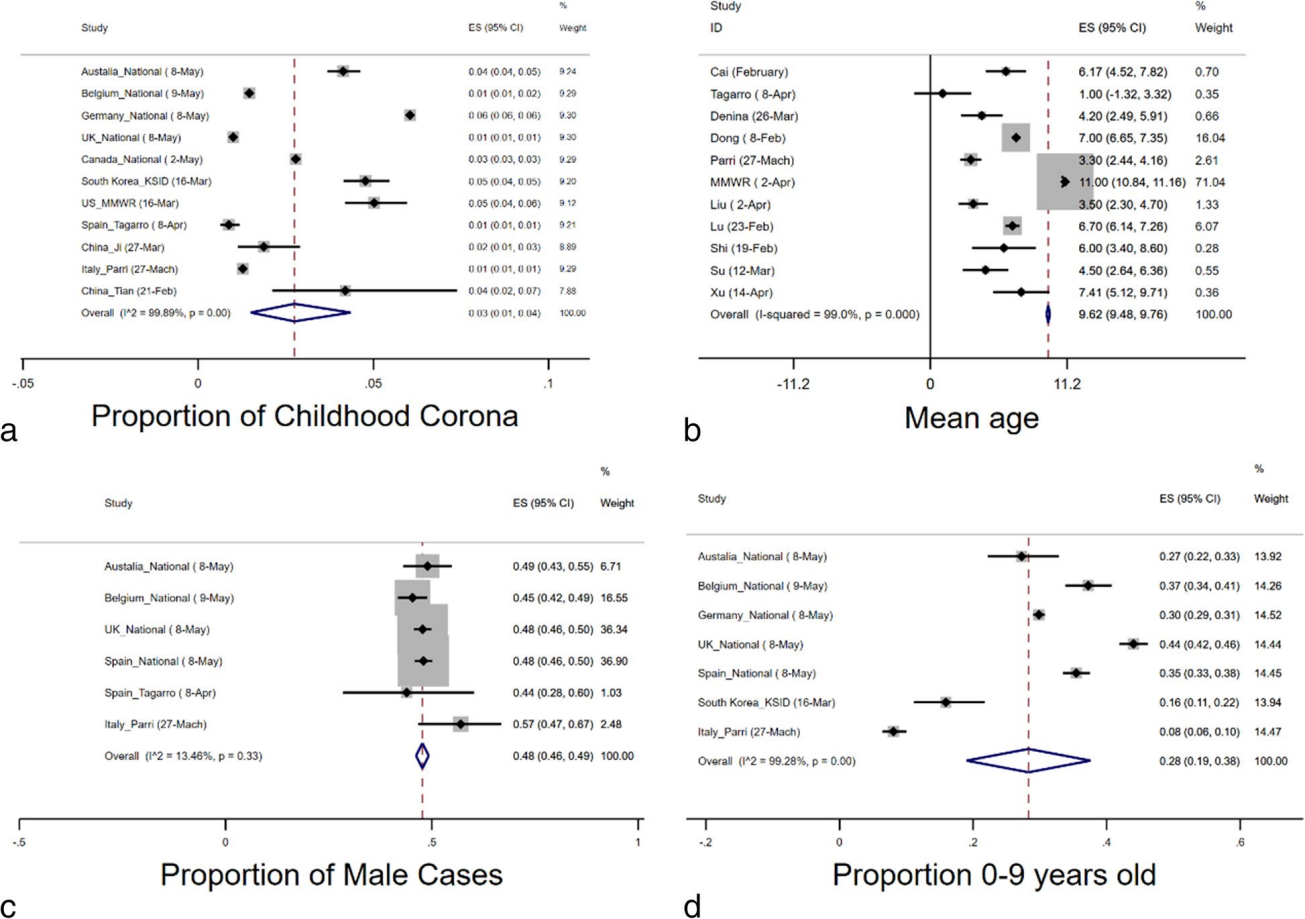
Combined Forest Plots for epidemiologic features of COVID-19 in children

Egger’s, Begg’s tests both showed no significant publication bias for the estimated proportion of pediatric cases (*p* = *0.105 and 0.449*, respectively). Publication bias funnel plots for analysis of the proportion of pediatric cases and mean age are shown in Figs. 2a and b. Heterogeneity assessed by I^2^ values for the proportion of pediatric cases (childhood corona and 0–9 years old), the proportion of male cases, and mean age is depicted in each of the corresponding forest plots (Figs. 3a, b, c and d).

### Prevalence of children's cases involvement and estimated mean age

The proportion of pediatric cases has been reported in 18 publications (Fig. 5a). The meta-analysis estimated an overall rate of involvement at 12% (95% CI: 9–15%) among children, with an I^2^ of 98.36%. Moreover, the meta-analysis estimated an overall proportion of male cases and mean age of 38% (95% CI: 24–53%) and 9.83 years (95% CI: 8.67–10.98), respectively (Figs. 3c and b).

**Fig. 4:**
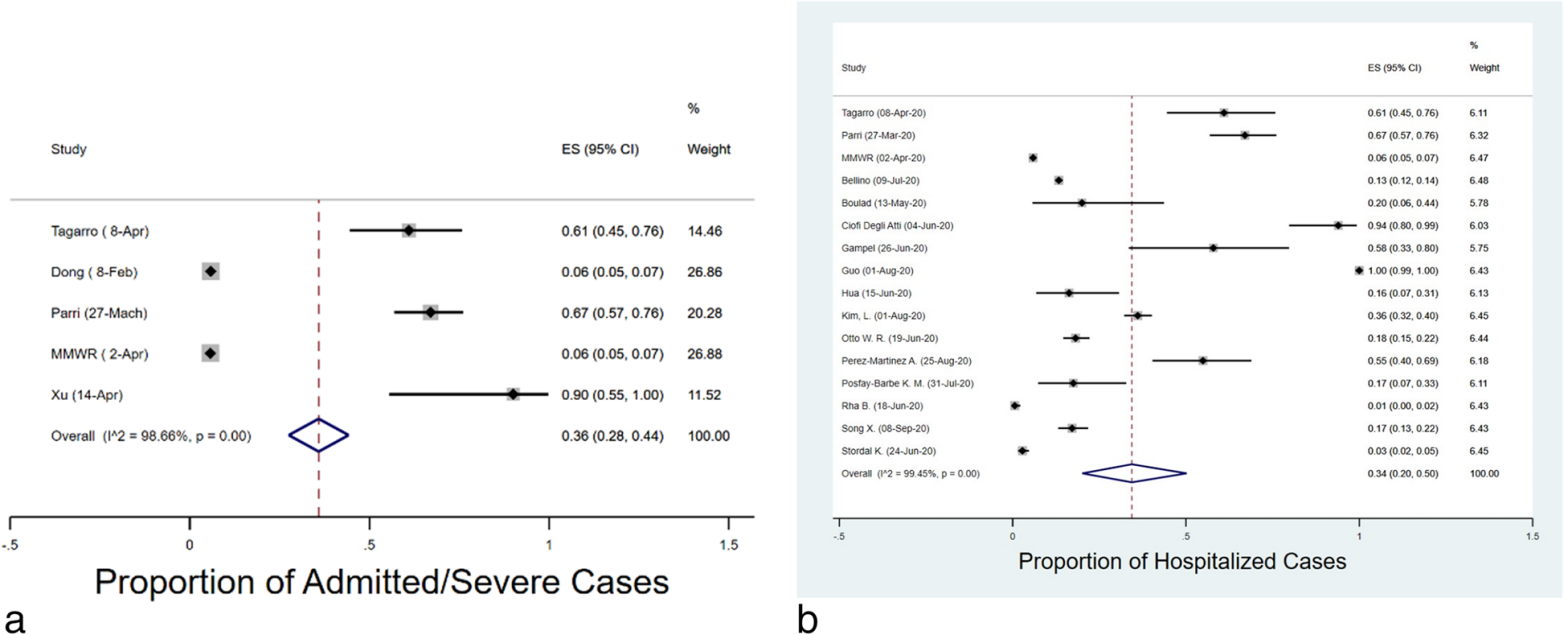
Combined Forest Plots for the hospitalized/childhood COVID-19 cases

### Household exposure and disease severity

**Fig. 5:**
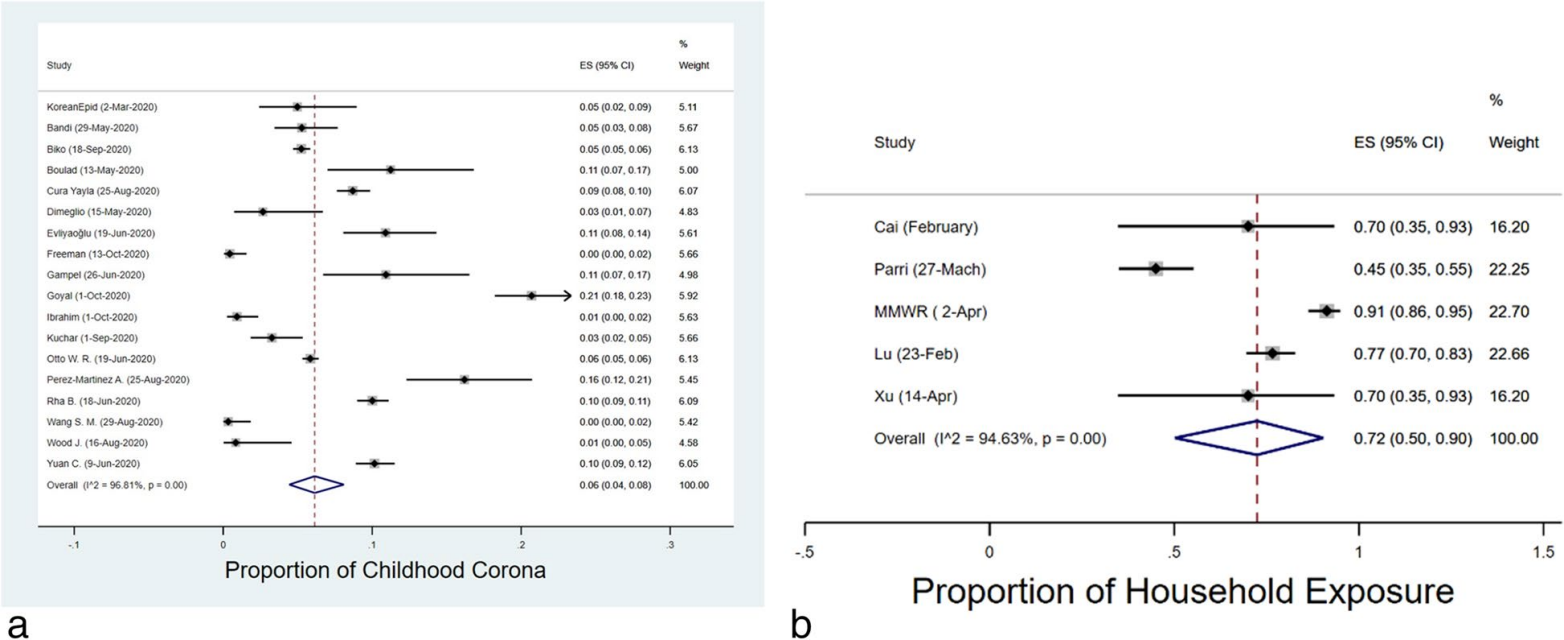
Forest plot for the proportion of pediatric COVID-19 cases and household exposure

The proportion of household exposure was calculated to be 50.99% (95% CI: 20.80%–80.80%) among the five studies that reported household exposure (Fig. 5b). The proportion of admitted cases was calculated to be 45% (95% CI: 24%–67%) among the 18 studies that reported the proportion of admitted cases (Figs. 4a and 4b). Heterogeneity (I^2^) statistic was 99.66% and 99.75%, respectively.

### Characteristics

**Fig. 6:**
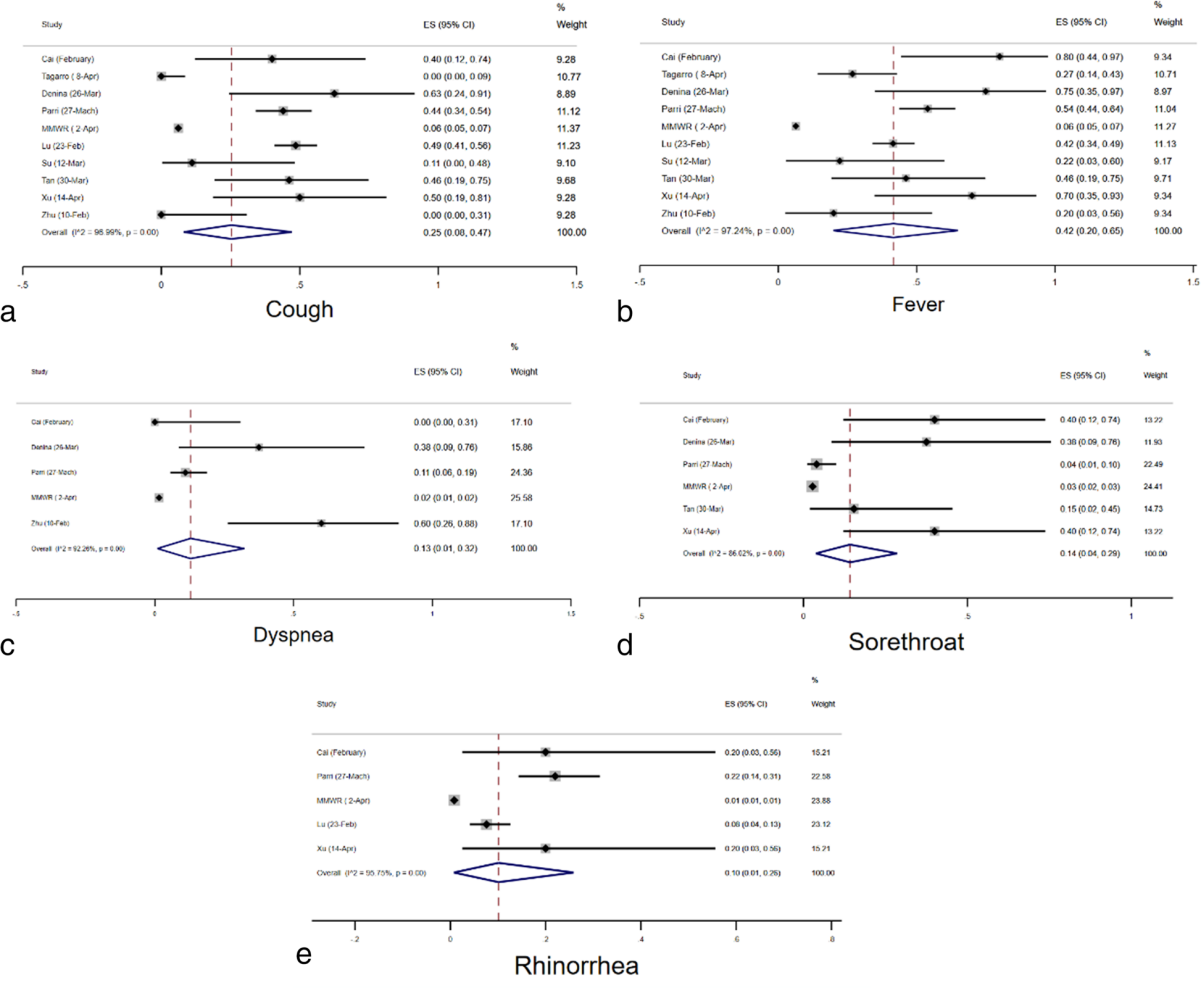
Combined Forest plots for the classical symptoms of COVID-19

**Fig. 7:**
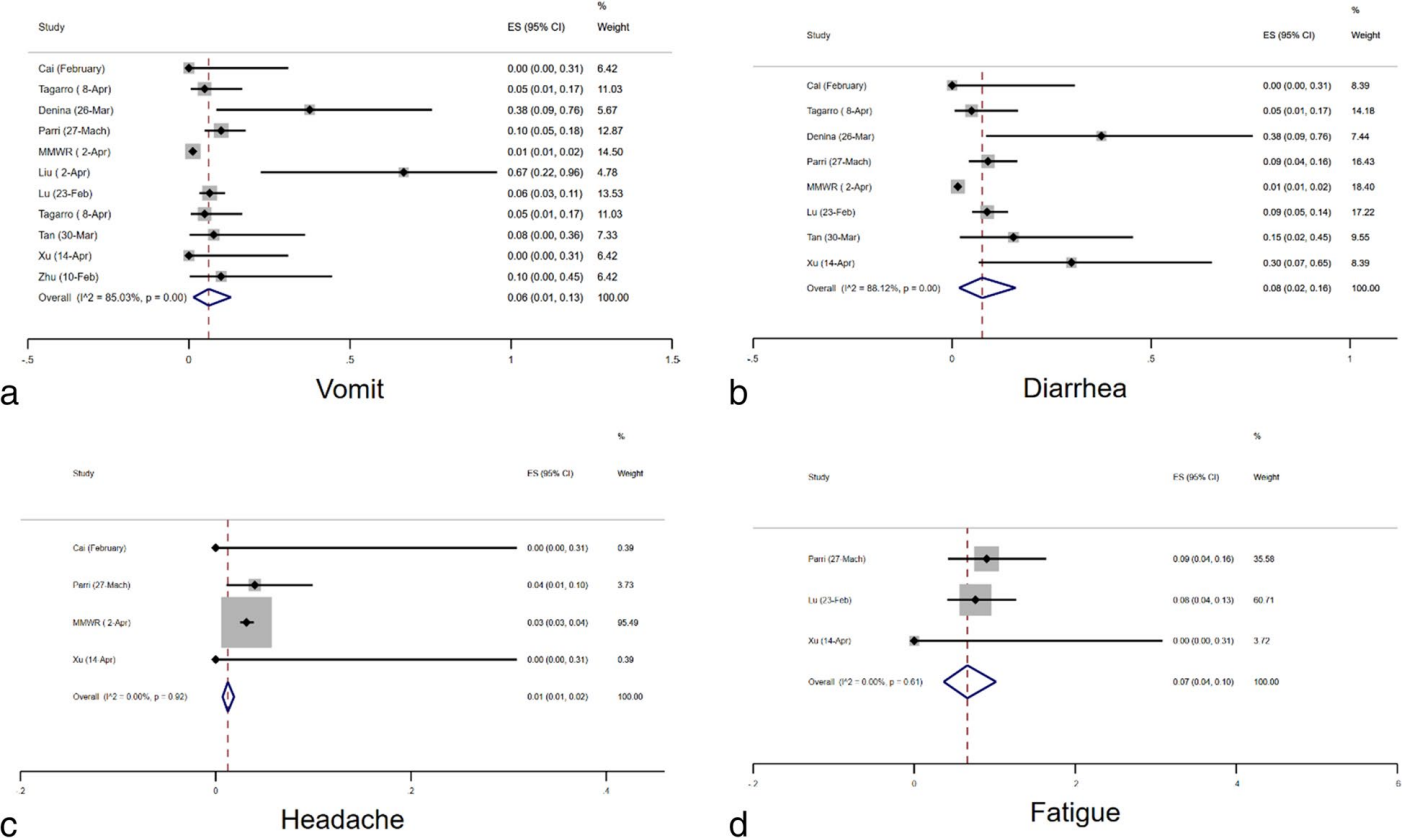
Combined Forest plots for the atypical symptoms COVID-19 pediatric cases

The prevalence of comorbidity was estimated to be 31% (95% CI: 0.16–0.48) according to the meta-analysis. The prevalence of cough, fatigue, fever and dyspnea was calculated to be 25% (95% CI: 0.16–0.36), 9% (95% CI: 0.03–0.18), 33% (95% CI: 0.21–0.47) and 9% (95% CI: 0.04–0.15), respectively (Figs. 6a, b, c, d and e). Furthermore, the meta-analysis estimated the prevalence of diarrhea, headache, rhinorrhea, sore throat and vomiting to be 6% (95% CI: 0.03–0.10), 9% (95% CI: 0.05–0.15), 13% (95% CI: 0.06–0.22), 7% (95% CI: 0.14–0.22) and 7% (95% CI: 0.02–0.14), respectively (Figs. 7a, b, c and d). Lastly, it is estimated that 4% (95% CI: 1–8%) of cases required Intensive Care Unit (ICU) admission according to the studies analyzed (Fig. 4a).

### Comment

#### Principal findings

Considering the proportion of children in the general population [24, 27], this in-depth review shows a low rate of SARS-COV-2 infection in children. The novel coronavirus affects both sexes similarly. There is an estimated high rate of household exposure and cross infection among family members, however, children have less chance of getting a severe form of the disease.

#### Strengths of the study

In this extensive review of the literature, pooled data of 743,763 infected patients were extracted by a systematic approach to collate the individual estimates reported from 4 continents all around the globe which gives a clear picture of the pandemic in the pediatric population. Potent statistical methods for meta-analysis with STATA software support our results.

## Discussion

This study aimed at describing the COVID-19 epidemiological characteristics in pediatric patients. The knowledge of COVID-19 in pediatric patients has improved exponentially since the inception of the disease in 2019 and so was cases. In the current investigation, we included a total of 671 studies from the primary search and effectively conducted a qualitative systematic review on 32 articles of which 27 (79.4%) articles were of good quality, with no significant publication bias. We found that the overall prevalence rate of COVID-19 among pediatric patients was 12% (95% CI: 9–15%). The frequency of pediatric COVID-19 increased to 19.0% as of 7 April 2022 in the US [45]. Moreover, this study found a higher prevalence of COVID-19 among female pediatric patients than males (38% (95% CI: 24–53%)), contrary to other studies which reported male dominance, such as Al Mansoori et al. (50.3%) [46], Dong Y et al. (56.6%) [47], and Armin S et al.(59.4%) [48]. The mean age of our population was 9.83 years while other studies reported a mean of 5.3 years [48]. Such variations may emanate from differences in study population and location. However, our population had a median age of 7 years, with a range from birth to 16 years, which was similar to previous studies [46, 47], indicating that children of all ages may be susceptible to COVID-19.

Our study found 50.99% of household exposure among the eight studies that reported household exposure. A previous investigation by Cheng-Xian et al. found that family clustering was a major (66%) transmission route for pediatric COVID-19 [49]. This study showed a high rate of household exposure before diagnosis in children later infected with COVID-19 (Fig. 5b) and bears an epidemiological significance indicating homes as the primary source of COVID-19 transmission in this period. However, Zhang C et al. (2020) found no direct evidence in their study regarding the transmission from children to adults [50]. Regardless, the risk of family cluster transmission from asymptomatic children should not be downplayed but be considered in policymaking for epidemic control. Moreover, preventive measures should be observed especially by family clusters of infections. High-risk children such as the family members of the confirmed cases, should take protective measures, may be recommended to take mineral supplements, high protein diets, fruits, and also pay attention to child psychological protection and intervention, as well as the living environment, etc. In addition, this study showed that children are more likely to present with diarrhea than adults, on top of respiratory symptoms and fever. Considering the high household rate of COVID-19 exposure for children, physicians are encouraged to investigate more into the family history and contacts of the child presenting with symptoms. It also signifies the importance of social-distancing the infected household members from children as a preventive measure.

As in all previous investigations, fever and cough were the main symptoms that could be associated with gastrointestinal symptoms such as nausea, vomiting, and diarrhea, and other symptoms like sore throat, dizziness, headache, and myalgia. The prevalence of cough, fatigue, fever, and dyspnea were 25%, 9%, 33%, and 9% respectively. Our study found the prevalence of cough (25%) between other studies which recorded 32.4% [49], (62%) [50], (67%) [51] and 19% [52]. Previously, Haiyan Q et al. reported a fever prevalence of 36% among pediatric patients [52], while other studies found 77.9% [49], (76%) [50], and (78%) [51] but the current study (33%) was below the average. Other prevalence parameters found through meta-analysis were diarrhea, headache, rhinorrhea, sore throat and vomiting to be 6% (95% CI: 0.03–0.10), 9% (95% CI: 0.05–0.15), 13% (95% CI: 0.06–0.22), 7% (95% CI: 0.14–0.22) and 7% (95% CI: 0.02–0.14), respectively. Gastrointestinal symptoms were mainly diarrhea (6%) and vomiting (7%) which were lower than previous studies vomiting (12%) [50] and diarrhea (12%) [50]. Headache was only prevalent in 9% of our study, however, some studies such as (57%) [51], report this symptom as major for pediatric COVID-19. Lastly, it is estimated that 4% (95% CI: 1–8%) of cases required ICU admission according to some studies.

Since many children do not show the classic picture of viral pneumonia, physicians must have a high level of suspicion not to miss COVID-19 in a child. Moreover, there have been some reports of unusual clinical presentations such as skin rash [16] and neurologic symptoms (upward gaze and spastic limbs) [20]. Given the different respiratory and GI presentations, and inconsistent reports of unusual presentations, a global registry is required, similar to what was done for pediatric cancers, and since the low rate of infection in children and lack of registry are serious limitations on the way of understanding disease characteristics in children.

Considering the high household rate of COVID-19 exposure for children, physicians are encouraged to investigate more into the family history and contacts of the child presenting with symptoms. It also signifies the importance of social distancing the infected household members from children. We were unable to perform a meta-analysis on the duration of incubation period or shedding of the virus in children but there are reports [1, 53] that both are longer in children about 6.5 days. Indeed, an undiagnosed child with COVID-19, in addition to being at risk of getting the severe form of the disease, could act as a significant source of infection transmission to others. Thus, we recommend policymakers emphasize standard infection control precautions and safety measures during this pandemic, such as physical distancing, wearing a mask, room ventilation, avoiding crowds, handwashing, and coughing into a bent elbow or tissue among children during home and schooling.

COVID-19 cases in children showing a systemic inflammatory syndrome have been reported in at least 8 states in the US (including California, Delaware, Louisiana, Massachusetts, New Jersey, New York, and Pennsylvania as well as Washington, D.C.) [54]. Currently, it is not known why children usually show mild to moderate forms of infection and only rarely present with cytokine storm syndrome [55]. Various hypotheses exist like fewer travels, less exposure to people (especially at the beginning of the pandemic), clearer respiratory tracts with better pathogen clearance compared to adults, fewer comorbidities, and strong innate and weaker adaptive immune defenses [56].

Fever is a prominent sign of both Kawasaki Disease (KD) and COVID-19 infection. Due to the COVID-19 pandemic, the first differential diagnosis to have in mind in a child with fever is probably COVID-19 infection or its combination. There are a few reports on this subject [57, 58]. KD is a rare disease with an estimated prevalence of 0.10% among children younger than 5 years old [59]. Its diagnosis is very important and missing it could result in complications like Coronary Artery Aneurysms (CAA). The recommendation is to be always suspicious of KD in a febrile child, especially if younger than a year old. If KD is diagnosed, intravenous immunoglobulin treatment should be immediately started, preferably in the first 7 days from the onset of fever [57, 58]. In the published literature only one case of COVID-19 infection in an infant with classic KD signs has been reported, who was discharged with Low-dose Aspirin pending further cardiologic evaluation [57]. Most recently, there have been some worrying reports of a presumed severe COVID-Kawasaki syndrome in the affected children from the US, UK, France, Italy, and Spain [60, 61]. It is believed to be related to poorer outcomes. There have been reports of 33 COVID-Kawasaki syndromes only in New York. Considering the low number of patients under 5 years old affected by COVID-19, the risk of Kawasaki disease likely is enormous. Kawasaki Disease was one of the first diseases where a strong Human Leukocyte Antigen (HLA) association was found [62]. Hence, this suggests certain HLA-types in children may be more susceptible to severe forms of SARS-COV-2 infection and could be an important clue to the etiology of poor prognosis of COVID-19 in this age group.

### Limitations of the data

We did not have access to certain published papers. Due to insufficient data, we were not able to perform a meta-analysis on some aspects of the disease such as the percentage of children with comorbidities, duration of the incubation period, and some symptoms like abdominal pain. Our investigation did not include laboratory and radiographic findings and we recommend future research into these. Future studies need to be performed on the COVID-Kawasaki syndrome to determine risk factors for poor outcomes in children. There is a possibility of publication bias in some reports, especially for mean age, and we recommend the creation of a global registry on pediatric COVID-19.

### Future recommendations and vaccination priority in children

This investigation described the characteristics of epidemiological characteristics of COVID19 in pediatric patients. Furthermore, the analyzed data from minors under the age of 19 shows that they are less prone to developing severe COVID-19 disease but are equally at risk of contracting the infection. Similar to previous investigations, the presentation of SARS-CoV-2 infection is somewhat different from the adult population. For example, children have longer incubation and shedding periods than adults, an epidemiological phenomenon that places pediatric age groups as potential hosts of the infection and capable of infecting many people. This evidence shows that children might be spreaders of COVID-19 more than we ever thought, and for the pandemic to stop, all children should be involved in mass preventive measures like vaccination programs, social distancing, and masking. In addition, it is encouraged to involve children in various clinical trials for COVID-19 vaccines and drugs to generate sufficient safety and efficacy profiles for this age group.

## Conclusion

Children are not safe from COVID-19 infection. About 37% of those diagnosed will need admission or end up with a severe form of the disease. The pediatric clinical picture of COVID-19 is not simply a classic respiratory infection but unusual presentations have been reported. Mortality among children with COVID-19 is rare. Given the high incidence, we propose a global registry on pediatric COVID-19 cases be developed to help identify disease characteristics, similar to what was done previously for pediatric cancer. Considering the potential of children to act as reservoirs and spread the coronavirus, we strongly recommend the inclusion of children in population-based preventive measures like vaccination as well as clinical trials to ensure efficacy, safety, and tolerability in this age group.

## Supplementary Information

**Additional file 1: Supplementary Table 1.** Quality assessment of the articles included in the final analysis of the study using the Newcastle-Ottawa Quality Assessment Scale (NOS). **Supplementary Figure 1.** Graphical representation of the quality assessment of included studies based on checklist items.

## Data Availability

The datasets used and/or analyzed during the current study are available from the corresponding author upon reasonable request.

## Abbreviations

CAA: Coronary Artery Aneurysms
ICU: Intensive Care Unit
KD: Kawasaki Disease
MMWR: Morbidity and Mortality Weekly Report
NOS: Newcastle–Ottawa Quality Assessment Scale
RNA: Ribonucleic Acid
SD: Standard Deviation
CONFIDENCE: Coronavirus Infection in Pediatric Emergency Departments
