# Supplementary material for: A systematic review and meta-analysis of the epidemiological characteristics of COVID-19 in children
Source: BMC Pediatr. 2022 Oct 22;22:613. doi: 10.1186/s12887-022-03624-4 (PMC9587668; doi:10.1186/s12887-022-03624-4)
Supplement: Supplementary file 1 — Additional file 1: Supplementary Table 1. Quality assessment of the articles included in the final analysis of the study using the Newcastle-Ottawa Quality Assessment Scale (NOS). Supplementary Figure 1. Graphical representation of the quality assessment of included studies based on checklist items. [file 12887_2022_3624_MOESM1_ESM.docx]

**Supplementary Table 1:** Quality assessment of the articles included in the final analysis of the study using the Newcastle-Ottawa Quality Assessment Scale (NOS).

| **ID** | **Study** | **Score** | **Quality Rating (Good, Fair, or Poor)** |
| --- | --- | --- | --- |
| 1 | Tagarro | 12 | Good |
| 2 | KoreanEpid | 12 | Good |
| 3 | Dong | 11 | Good |
| 4 | Parri | 12 | Good |
| 5 | MMWR | 13 | Good |
| 6 | Tian | 10 | Good |
| 7 | Bandi | 8 | Fair |
| 8 | Bellino | 11 | Good |
| 9 | Biko | 11 | Good |
| 10 | Boulad | 10 | Good |
| 11 | Ciofi Degli Atti | 7 | Poor |
| 12 | Cura Yayla | 12 | Good |
| 13 | Dimeglio | 7 | Poor |
| 14 | Evliyaoğlu | 8 | Fair |
| 15 | Freeman | 11 | Good |
| 16 | Gampel | 10 | Good |
| 17 | Goyal | 12 | Good |
| 18 | Guo | 12 | Good |
| 19 | Hua | 12 | Good |
| 20 | Ibrahim | 12 | Good |
| 21 | Kim, L. | 12 | Good |
| 22 | Kuchar | 12 | Good |
| 23 | Otto W. R. | 8 | Fair |
| 24 | Perez-Martinez A. | 7 | Poor |
| 25 | Posfay-Barbe K. M. | 10 | Good |
| 26 | Rha B. | 9 | Fair |
| 27 | Song X. | 11 | Good |
| 28 | Stordal K. | 12 | Good |
| 29 | Swann O. V. | 11 | Good |
| 30 | Wang S. M. | 9 | Fair |
| 31 | Wood J. | 10 | Good |
| 32 | Yuan C. | 11 | Good |
